# Number of Players and Relative Pitch Area per Player: Comparing Their Influence on Heart Rate and Physical Demands in Under-12 and Under-13 Football Players

**DOI:** 10.1371/journal.pone.0127505

**Published:** 2016-01-11

**Authors:** Julen Castellano, Asier Puente, Ibon Echeazarra, Oidui Usabiaga, David Casamichana

**Affiliations:** 1 University of the Basque Country (UPV/EHU), Vitoria-Gasteiz, España; 2 Gimbernat-Cantabria University School associated with the University of Cantabria (UC), Santander, España; Universidad Europea de Madrid, SPAIN

## Abstract

The aim of the present study is to analyse the influence of different large-sided games (LSGs) on the physical and physiological variables in under-12s (U12) and -13s (U13) soccer players. The effects of the combination of different number of players per team, 7, 9, and 11 (P7, P9, and P11, respectively) with three relative pitch areas, 100, 200, and 300 m^2^ (A100, A200, and A300, respectively), were analysed in this study. The variables analysed were: 1) global indicator such as total distance (TD); work:rest ratio (W:R); player-load (PL) and maximal speed (V_max_); 2) heart rate (HR) mean and time spent in different intensity zones of HR (<75%, 75–84%, 84–90% and >90%), and; 3) five absolute (<8, 8–13, 13–16 and >16 Km h^-1^) and three relative speed categories (<40%, 40–60% and >60% V_max_). The results support the theory that a change in format (player number and pitch dimensions) affects no similarly in the two players categories. Although it can seem that U13 players are more demanded in this kind of LSG, when the work load is assessed from a relative point of view, great pitch dimensions and/or high number of player per team are involved in the training task to the U12 players. The results of this study could alert to the coaches to avoid some types of LSGs for the U12 players such as: P11 played in A100, A200 or A300, P9 played in A200 or A300 and P7 played in A300 due to that U13>U12 in several physical and physiological variables (W:R, time spent in 84–90%HR_max_, distance in 8–13 and 13–16 Km h^-1^ and time spent in 40–60%V_max_). These results may help youth soccer coaches to plan the progressive introduction of LSGs so that task demands are adapted to the physiological and physical development of participants.

## Introduction

Competition formats in junior soccer categories must be adapted to the conditions of the players, which frequently results in alterations to the regulations in order to adapt the response to the child’s condition [1]. Association football, in line with other sports, has been modified to make playing sport easier for children and youngsters [2]. However, a lack of consensus means that different competitive formats are established which are applied to different age categories [3], without sufficient scientific cross-checking [4] to provide information about the effects which different competitive formats or large-sided games (LSGs) may have on young players in a training context. Over the last few years, some studies [5, 6] have examined the effects of structural adaptations of the game on players and teams by changing the pitch size, type of ball, the number of players per team or the duration of the match [2].

It is well known that, in terms of performance, modification of the number of participating players per team affects task intensity [7]. Therefore, the influence of this relative area per player variable has been researched in various pieces of work [8], from 1 on 1 drills [9] to 8 on 8 [9, 10], along with other intermediate compositions [8,11,12]. It has been observed that game formats with fewer players involve less total distance covered, increased heart rate (HR), lactate concentration and subjective perception of effort (RPE), this being partly explained by the increase in ball play [10, 13, 14]. On the other hand, a greater demand for high speed runs and sprints could occur in situations with more players [10,12], although it is not known whether the influence of player number variation in LSG situations is dependent on the age of the participants.

Other research has studied how modifying pitch dimensions affects the demands on players [15, 16, 17, 18], with these being altered both in absolute and relative terms [8]. Scientific literature, although inconclusive, supports the idea that the bigger the pitch size, the greater the physical and physiological intensity [15, 17, 19]. Furthermore, pitch size has an influence on individual actions, with a higher frequency of dribble control and shooting control and a higher number of interceptions and clearances in more reduced spaces [15]. It also has an effect on the collective behaviour of the teams [20, 21], as they develop particular strategies depending on the pitch size, to help them carry out the task in the most efficient way. However, it is not known whether modifications in pitch size have the same effect on different age categories.

Thanks to the increased accessibility of modern technology, a wider body of information is available concerning the physical demands imposed on young players and, consequently, on the adaptation of training drills, starting from, above all, the study of small-sided games [11, 22], and competition matches, whose characteristics are closer to LSG formats. The study of competitions with junior players has allowed physical-physiological profile comparisons to be made between players of different ages [23, 24, 25, 26]. This has been done to discover whether there are any differences between players selected by their clubs and those which are not [3], or to relate physical performance in competition with different specific intermittent tests [27, 28]. Thus, for example [3] compared the distances covered in a competitive format of 6-a-side on a pitch measuring 45 m x 26 m (97 m^2^ per player) in U9 and U10 players. The authors found that, despite the maturity of the players, they did not cover a significantly greater distance per match minute. However, older players covered a significantly smaller distance at lower rates of movement, and a bigger distance at high movement and sprint speeds. Other works [23, 24, 25] have chosen to put the physical and physiological demands on the players into perspective on the basis of individual maximums [29], when they monitored football players between 13 and 18 years old, as had been proposed previously. The results indicated that the younger players covered a greater distance in superior running speed ranges, but there were no differences in intensity as measured by heart rate.

Previous research leaves various avenues unexplored, whilst the results may depend on the particular characteristics of the sample or the variation in the selected competitive format, in relation to match format, pitch size or match length. Regarding this last point, there were notable differences between age groups, making comparisons difficult [26], because U12, U13, U14 and U15 players played three 25 min sessions, or two 25 min sessions plus two 12.5 min sessions, whilst the U16s played two 40 min sessions. Finally, in spite of the ecological validity of the study of players’ physical activity in competition itself, the influence of tactical and strategic factors on physical performance during matches as well as contextual variables such as place, score or opponent level could add an unwanted bias for the researcher, in an attempt to discover in detail the imposed demands [30]. Consequently, discovering the demands of LSG formats, using the same players and standardizing possible extraneous variables (home/away, match length, score…) which may affect the players’ response, could present an ideal opportunity to discover how far these demands are affected by the modification of certain match variables. LSG formats are habitually characterized by being played on large pitches (≥200 m^2^) with a high number of players per team (≥7), similar to competition matches.

In this sense, the authors are unaware of any work which has compared the physical demands on players from two age groups, using different LSGs for both, in the same conditions, avoiding, wherever possible, variability inherent to competition. It is for this reason that the aim of this work is to study heart rate response and locomotor activity in U12 and U13 players in different LSGs, altering both the number of participants per team (7, 9 and 11) and the relative playing space per player (100, 200 and 300 m^2^), whilst keeping the duration of both constant. The results of this work will provide further information regarding the demands produced by different LSGs on junior football players. That is, to provide information with which to assess the most appropriate pitch dimensions and number of players per team in each age category, so that the conditioning factor does not represent a limitation for the development of their game.

## Methods

### Participants

The participants in the study were U12 (n = 22, age: 12.1 ±0.4 years, height: 147.5±6.0 cm, body mass: 38.9±5.4 kg, peak speed: 23.2 ±1.0 Km h^-1^) and U13 (n = 22, age: 13.3±0.5 years, height: 152.9±5.7 cm, body mass: 42.2±5.2 kg, peak speed: 25.1 ±0.3 Km h^-1^) outfield players from a Spanish Premier League Academy. They had an average of three years’ (U13) and at least one year’s (U12) experience in soccer training and competition in the same club prior to the study.

The players generally participated in three (U12) or four (U13) technical/tactical training sessions (each lasting around 75 min for U12 or 90 min for U13 per training session), in addition to a competitive match per week during the season. They played on a regular sized football field (60 m x 100 m) with the same rules of eleven-a-side football. In order to avoid potential imbalances between the two teams, and to ensure their equivalence, the players were classified following a subjective appraisal by the coach. When the matches were between nine-a-side teams, the demarcations of seven-a-side matches were respected (with a team formation of one goalkeeper, three defenders, three mid-fielders and one striker) with the addition of one mid-fielder and one striker. For eleven-a-side games, one mid-fielder and one striker were added to the nine-a-side formation (1-4-3-3 formation). The same players were monitored for HR and locomotor activity in the different LSGs. The total number of measurements was 180, that is, 10 measurements in each LSG format (P7/A100, P9/A100, P11/A100, P7/A200, P9/A200, P11/A200, P7/A300, P9/A300 and P11/A300) and in each age (U12 and U13).

### Ethics Statement

All the players were notified of the research design and its requirements, as well as the potential benefits and risks, and each player’s parent, guardian or care-giver signed the consent form prior to the start of the study. It was explained to the players both verbally and in writing that they would be free to withdraw from the study without giving any reasons and without incurring any penalties regarding their academic position. The Ethics Committee of the University of the Basque Country (CEISH) also gave its institutional approval for the study.

### Physical performance: global indicators

Physical performance was measured using a portable GPS device operating at a sampling frequency of 10 Hz (MinimaxX v.4.0, Catapult Innovations). The data was then downloaded to a PC and analysed using the software package Logan Plus v.4.5.1 (Catapult Innovations, 2010). This technology has previously been shown to be a reliable and valid way of monitoring high-intensity running [31, 32].

The global performance indicators were as follows: total distance covered (TD); work:rest ratio (W:R), defined as the distance covered by the player at a speed ≥4 km h^–1^ (period of activity or work) divided by the distance covered at a speed <4 km h^–1^ (period of recovery or rest); and player load (PL), which was determined via accelerometry [33, 34, 35], using a 100 Hz triaxial accelerometer that combined the accelerations produced in three planes of body movement. Player load is an indicator that seems to be highly correlated with the Edwards method and session-RPE [34], and the high reliability of its results, both within and between devices, suggests that accelerometers are able to detect changes or differences in physical activity [35].

### Physical variables: absolute and relative speed ranges

Speed ranges were assessed in two ways, using GPS devices. Firstly, and in line with previous studies [27, 36], we established five speed categories (all in km h^–1^): 0–3, 3–8, 8–13, 13–16, and >16. Secondly, as proposed by Buchheit et al. [37], we also estimated the distance covered at speeds relative to the maximum individual speed (V_max_) achieved during the speed test, on the basis of which three categories were established (all in km h^–1^): <40%, 40–60%, and >60% of the V_max_. The distance covered (in metres) in each one of these speed categories (both absolute and relative) was tracked. Similar relative ranges have previously been used [25].

### Physiological variables: mean and different heart rate intensity ranges

The physiological profile was assessed on the basis of HR [38], which was recorded every 5 seconds using a telemetric device (Polar Team Sport System, Polar Electro Oy, Finland). The individual maximum HR (HR_max_) of each player was determined by means of the Yo-Yo Intermittent Recovery Test-level 1 (YYIRT1) [39, 40] enabling four intensity zones to be established [8, 11]: <75%HR_max_, 75–84%HR_max_, 84–90%HR_max_, and >90%HR_max_. For the purposes of analysis the variables used were: percentage of time spent in each intensity zone during each LSG, and the percentage of the mean value with respect to the maximum HR obtained in the YYIRT1 (%HR_mean_).

### Assessment of large-sided games (LSGs)

Nine LSGs were used as independent variables: 1) the relative pitch area per player (A): 100 m^2^, 200 m^2^, or 300 m^2^ (A100, A200, and A300, respectively); and 2) the number of players (P) per team: 7, 9, or 11 (P7, P9, and P11, respectively) being the goalkeepers within the team. Although the overall pitch size was varied, the length:width ratio was held constant. The standard rules of 11-a-side soccer were followed in all LSGs.

### Procedure

The study was conducted over a five-week period (October-November) during the competitive season. In the weeks prior to this, the players were familiarised with all the LSG types and the material to be used. During the week immediately before the study began, each participant performed the YYIRT1 [40] in order to determine HR_max_. As in other studies [37] the maximal sprinting speed was also determined, which was assessed over 30 m, using photocells (Kit Racetime2 SF, Microgate, Italy). The order of the tests was firstly speed and secondly the YYIRT1. All procedures were carried out on the same day on an outdoor artificial pitch with the players wearing football boots.

For each team, nine training sessions were held (two per week, except for week 5) on an outdoor artificial grass pitch and at similar times of day. Each session began with a 15-min standard warm-up, followed by one of the LSGs played over two 12-min halves, with a 5-min passive rest period at half-time. The players did not take part in any intense physical activity in the 48 hours prior to the tasks.

During all the training sessions, coaches were present in order to offer encouragement to the players [19]. In addition, eight footballs were distributed around the edge of the pitch in order to maximize the effective playing time [15]. All the matches were played at the same time of day in order to avoid the effects of circadian rhythms on the results [41]. All participants were advised to follow a normal diet and to eat at more or less the same time of day (14:30 hours), with special emphasis being placed on a high intake of water and carbohydrates.

### Statistical analysis

The data is presented as means and standard deviations (means±SD). The variables did not fulfil the assumption of normality. In the event that a significant difference was observed, a two-way comparison was performed using the Mann-Whitney U test, with post hoc Bonferroni correction. Effect sizes were also calculated [42], and defined as follows: null, <0.3; mild, 0.3–0.5; moderate, 0.5–0.7; strong, 0.7–0.9; and very strong, 0.9–1.0. All the statistical analyses were performed using SPSS 19.0 for Windows (SPSS Inc., Illinois USA), with significance being set at *p*<0.05.

## Results

### Global Indicators

Table 1 shows the results for the global indicators. In TD significant differences are only observed in P11/A300 (U12<U13, p = 0.032, ES = 0.48). The W:R variable shows significant differences between categories in P7/A300 (U13>U12, p = 0.040, ES = 0.70) and in P11/A300 (U13>U12, p<0.001, ES = 0.70). The PL variable yields significant differences in: P7/A200 (U12>U13, p<0.01, ES = 0.54), P9/A100 (U12>U13, p<0.01, ES = 0.70), P9/A200 (U12>U13, p<0.01, ES = 0.71) and P9/A300 (U12>U13, p<0.01, ES = 0.53). Significant differences are not apparent between the two categories in V_max_.

**Table 1:** Mean and standard deviation (±SD) for the variables TD (total distance covered), V_max_ (maximum speed), PL (player load) and W:R (work:rest ratio) in relation to each of the nine large-sided games.

Players per team		Relative pitch area per player					
		100		200		300	
	Variables / levels	U12	U13	U12	U13	U12	U13
7	TD (m)	1718±150	1816±155	2067±127	2085±153	2186±90	2307±212
	V_max_ (km h^-1^)	20.4±2.2	19.4±1.6	21.7±1.9	21.9±2.0	21.9±1.3	22.4±0.8
	PL (UA)	283±26	267±47.5	330±39*	285±30	297±25	300±41
	W:R	0.2±0.1	0.3±0.1	0.4±0.1	0.4±0.1	0.4±0.1	0.6±0.1*
9	TD (m)	1867±126	1845±141	2075±147	2003±102	2159±183	2250±107
	V_max_ (km h^-1^)	21.1±1.7	20.5±1.5	21.3±1.6	21.7±2.0	21.4±1.6	22.1±1.4
	PL (UA)	297±35*	233±29	327±52*	246±23	310±35*	271±26
	W:R	0.3±0.1	0.3±0.1	0.4±0.1	0.4±0.0	0.4±0.1	0.5±0.1
11	TD (m)	1844±254	1766±181	1923±238	2148±212	2168±127	2314±134*
	V_max_ (km h^-1^)	20.6±1.7	19.6±2.1	20.8±1.2	21.9±1.5	21.5±2.4	22.6±1.9
	PL (UA)	257±55	229±49	241±41	274±59	285±41	306±39
	W:R	0.3±0.1	0.3±0.1	0.3±0.1	0.5±0.1	0.4±0.1	0.6±0.1*

Note: 100, 200, and 300 represent, respectively, the relative pitch areas of 100 m^2^, 200 m^2^, and 300 m^2^ per player, while 7, 9, and 11 correspond to the number of players per team (7, 9, and 11, respectively). U12 is players aged under 12 years and U13 under 13 years.

*Significant difference between categories p<0.05.

### Relative heart rate data and percentage of time spent in different heart rate intensity zones

**Fig 1:**
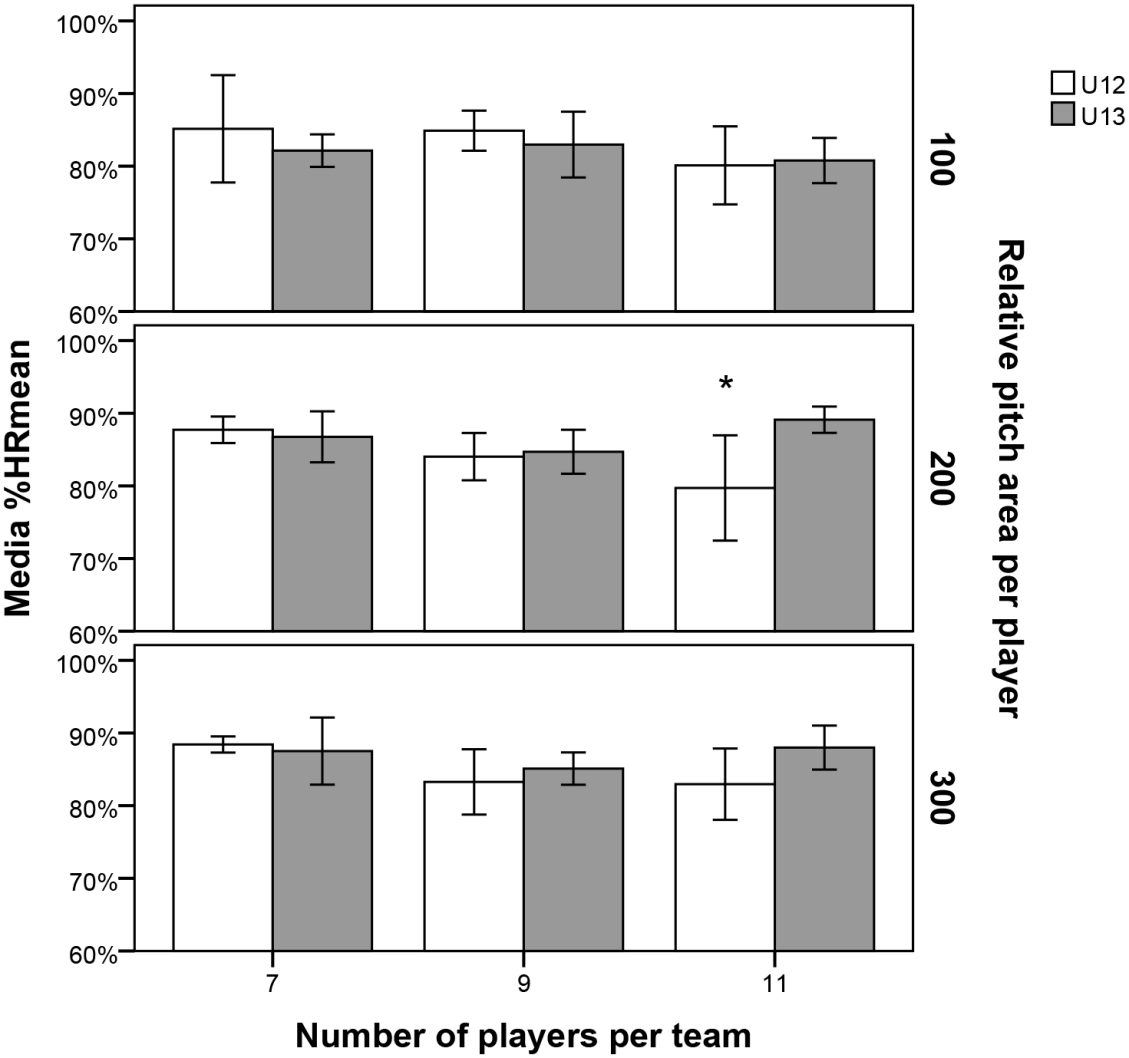
Mean and standard deviation (±SD) for the mean heart rate with respect to the individual maximum (%HR_mean_) for each of the nine competitive formats. Note: 100, 200, and 300 represent, respectively, the relative pitch areas of 100 m^2^, 200 m^2^, and 300 m^2^ per player, while 7, 9, and 11 correspond to the number of players per team (7, 9, and 11, respectively). *Significant difference between categories p<0.05.

Fig 1 presents the results in relation to %HR_mean_ (%), where significant differences are only seen in P11 with an area per player of 200 m^2^ (P11/A200). U12 players obtained significantly lower values than U13 (U12 = 79.7±8.7% and U13 = 89.1±1.7%, p = 0.018, ES = 0.57).

**Fig 2:**
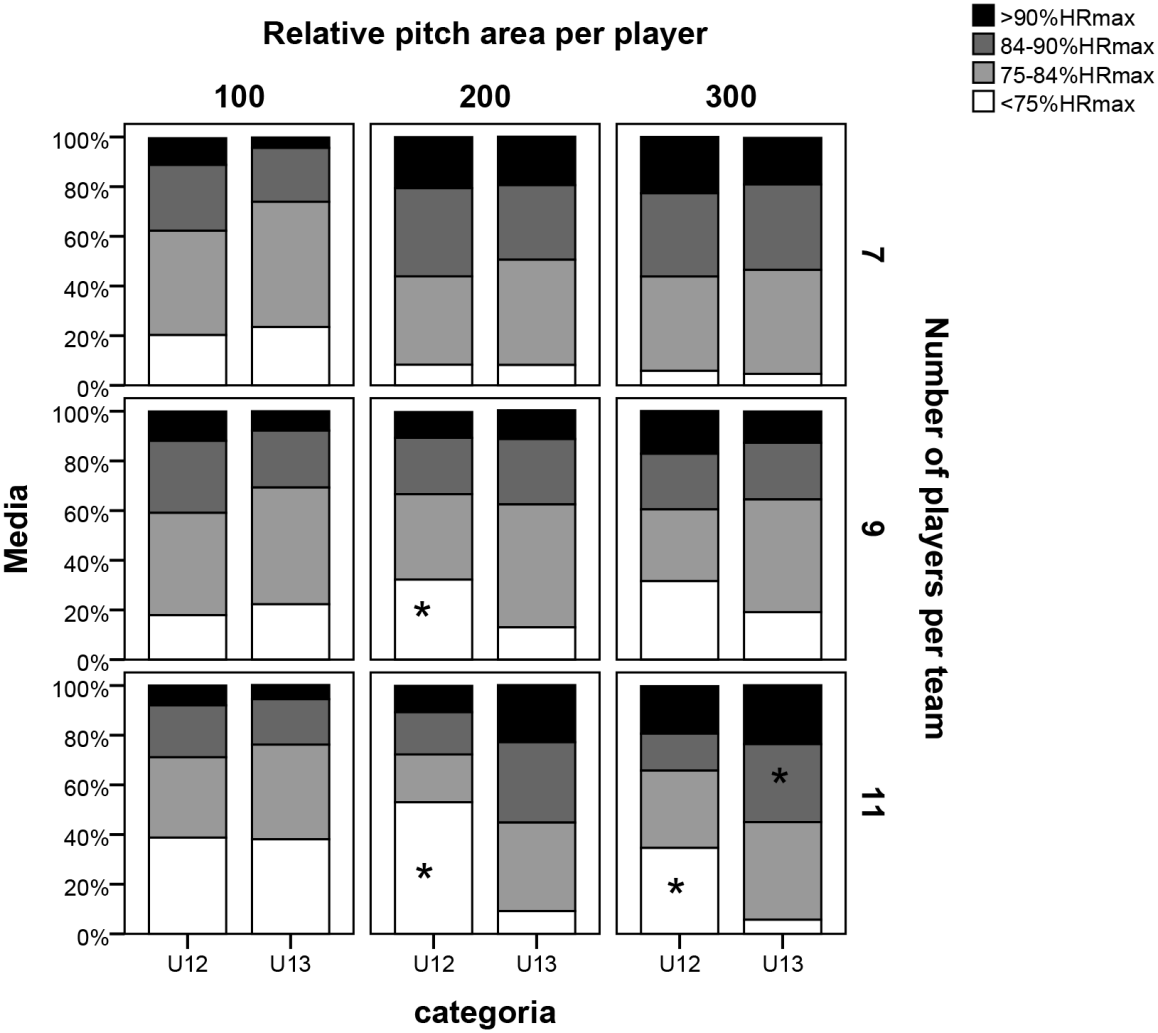
Percentage time spent in each heart rate intensity zone for each of the nine large-sided games. Note: 100, 200, and 300 refer to the relative pitch area (in m^2^) per player, while 7, 9, and 11 correspond to the number of players per team. *Significant difference between categories p<0.05.

Fig 2 shows the percentage time that players spent in different heart rate intensity zones. Signifcant differences in the time spent in each heart rate intensity zone are not apparent between U12 and U13 players in seven-a-side formats (P7), irrespective of the relative pitch size. However, the U12 players spent a considerably greater amount of time than the U13s in the zone <75%HR_max_ in P9/A200 (U12 = 32.2±24.0 vs. U13 = 13.0±11.1, p = 0.040, ES = 0.45), P11/A200 and P11/A300 (U12 = 53.0±39.1 vs. U13 = 9.2±9.0, p = 0.016, ES = 0.61 and U12 = 34.6±29.0 vs. U13 = 5.7±4.5, p = 0.026, ES = 0.57), whilst the U13s spent more time in the category of 84–89%HR_max_ in P11/A300 (U12 = 14.9±11.3 vs. U13 = 31.4±6.7, p = 0.040, ES = 0.11).

### Distance covered in absolute speed ranges

**Fig 3:**
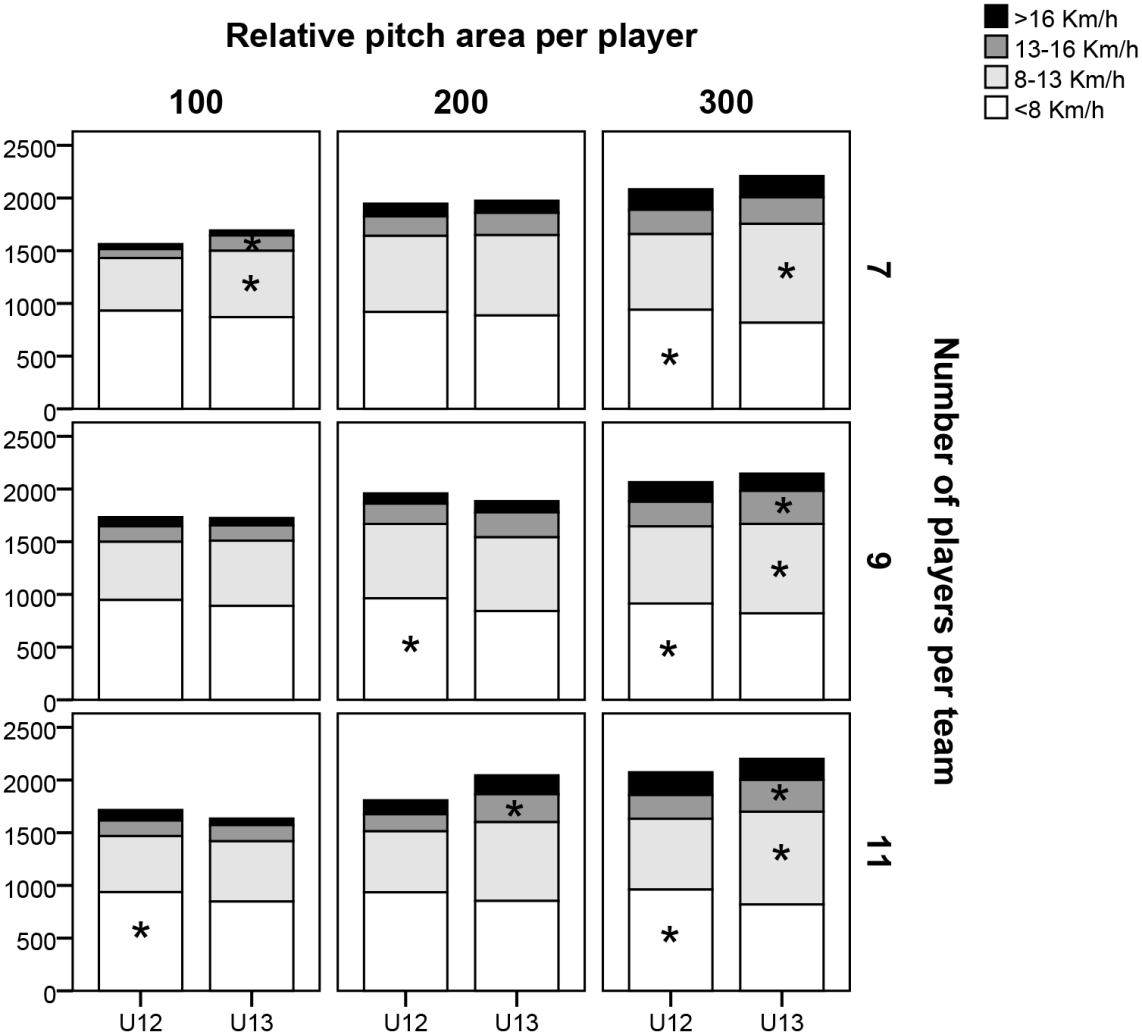
Distance covered in different absolute speed ranges in relation to the nine large-sided games. Note: 100, 200, and 300 refer to the relative pitch area (in m^2^) per player, while 7, 9, and 11 correspond to the number of players per team. *Significant difference between categories p<0.05.

Fig 3 shows the distance covered in relation to the different absolute speed ranges, where significant differences can be seen in the lowest speed category (<8.0 km h^-1^) in P7/A300 (U12 = 1043.3±58.8 vs. U13 = 916.3±76.3; p = 0.005, ES = 0.68), in P9/A200 (U12 = 1078.9±57.5 vs. U13 = 960.2±84.4, p = 0.002, ES = 0.63), in P9/A300 (U12 = 1007.5±73.9 vs. U13 = 924.5±39.8, p = 0.006, ES = 0.57), in P11/A100 (U12 = 1064.8±48.5 vs. U13 = 978.9±80.3, p = 0.017, ES = 0.54) and P11/A300 (U12 = 1055.3±66.9 vs. U13 = 932.7±65.6, p = 0.001, ES = 0.68). In the 8.0–13.0 km h^-1^ speed category there are significant differences in P7/A100 (U12 = 500.0±90.4 vs. U13 = 629.6±103.5, p = 0.05, ES = 0.55) and P7/A300 (U12 = 717.2±94.1 vs. U13 = 937.8±135.9, p = 0.005, ES = 0.69). In P9/A300 (U12 = 731.5±124.6 vs. U13 = 848.2±79.9, p = 0.023, ES = 0.48) and P11/A300 (U12 = 671.4±54.6 vs. U13 = 879.5±82.0, p<0.01, ES = 0.83). In the speed category 13.0–16.0 km h^-1^, differences can be observed in P7/A100 (U12 = 84.0±18.3 vs. U13 = 145.0±32.1, p = 0.004, ES = 0.76), in P9/A300 (U12 = 234.0±59.8 vs. U13 = 313.5±60.0, p = 0.008, ES = 0.55), P11/A200 (U12 = 161.8±80.5 vs. U13 = 264.7±60.6, p = 0.023, ES = 0.58) and P11/A300 (U12 = 225.4±54.3 vs. U13 = 301.9±87.5, p = 0.047, ES = 0.46).

### Distance covered in relative speed ranges

**Fig 4:**
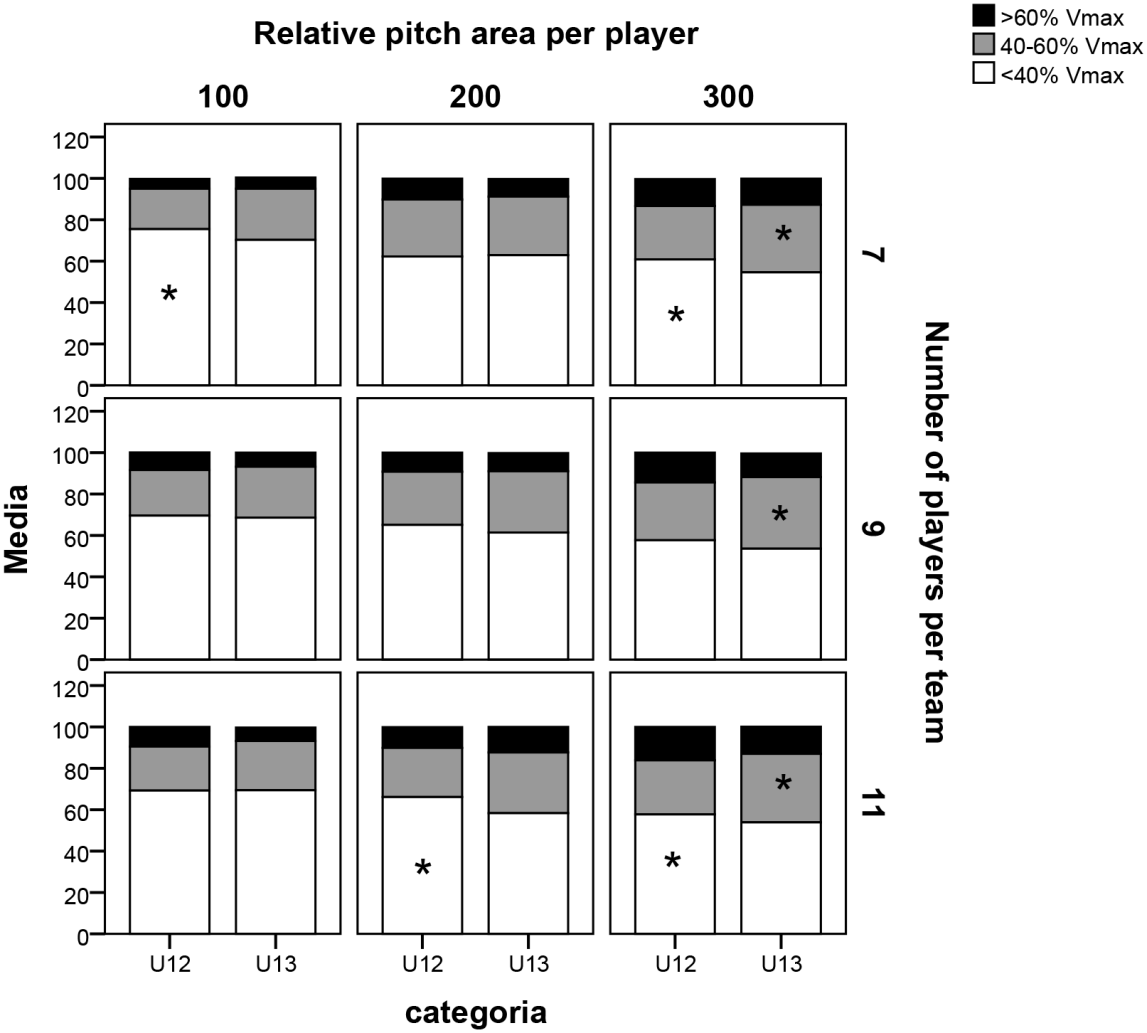
Distance covered in different absolute speed ranges in relation to the nine large-sided games. Note: 100, 200, and 300 refer to the relative pitch area (in m^2^) per player, while 7, 9, and 11 correspond to the number of players per team. *Significant difference between categories p<0.05.

Fig 4 shows the percentage of time which the players remained in the different relative speed ranges. Significant differences were found between the two categories in the following speed ranges: a) U12>U13 for the variable <40%Vmax in P7/A100 (U12 = 89.8±1.7 vs U13 = 86.9±2.4, p = 0.038, ES = 0.57), in P7/A300 (U12 = 80.7±3.7 vs. U13 = 75.6±4.4, p = 0.043, ES = 0.53), in P11/A200 (U12 = 84.5±3.8 vs U13 = 79.54.8, p = 0.05, ES = 0.50) and in P11/A300 (U12 = 78.6±0.9 vs. U13 = 75.7±3.2, p = 0.019, ES = 0.53); b) U12<U13 for the variable 40–60%Vmax in P7/A300 (U12 = 14.2±2.6 vs. U13 = 19.3±3.3, p = 0.01, ES = 0.65), in P9/A300 (U12 = 15.6±3.2 vs. U13 = 19.3±2.5, p = 0.01, ES = 0.54), and in P11/A300 (U12 = 15.0±1.4 vs U13 = 18.9±3.1, p = 0.004, ES = 0.63).

## Discussion

The aim of this work was to compare the same training drills in two different age categories (U12 vs. U13), by studying heart rate response and locomotor activity in players participating in different LSGs, altering both the number of players per team (7, 9 and 11) and the relative pitch area per player (100, 200 and 300 m^2^). The main conclusion drawn from the study was that a change in game format, whilst respecting some similar tendencies, implies physical and physiological demands in different proportions depending on the age of the players. To be precise, drills of smaller dimensions with fewer players per team (P7 and A100, P7 and A200, P9 and A100), produced practically no differences in physical and/or physiological intensity between age categories. However, as the number of players is increased to 9, and especially 11, and/or the area dimensions also (P9 and A300, P11 and A200 and above all P11 and A300), the game dynamic of younger players could be limited. This is as a result of them showing significantly lower values than older players in those variables which reveal intensity of play, such as: time spent in the range 84–90%HR_max_, distance covered >8Km h^-1^ or percentage of time spent in the variable 40–60%V_max_. This lack of intensity is probably due to the fatigue experienced by younger players in the second halves [25] when playing in big game formats on large pitches, above all for the U12s. This goes against the idea that it is preferable to acquire new abilities in situations without fatigue [43].

It should be pointed out that, from the outset, we must be cautious when comparing this current study with previous ones, due to, among other things, the sampling frequency of the GPS devices used. Whilst this work had a sampling frequency of 10 Hz, previous studies had a lower sampling frequency, with this being different between the various studies (*p*.*e*. 1 Hz in [3, 23, 24, 25] and 5 Hz in [26]). This could affect the results, above all those related to variables which cover high rates of movement [32].

As far as global indicators are concerned, it is worth highlighting that the PL variable, which provides information about accumulated accelerations and decelerations carried out by the players in the three planes of movement, yielded most of the differences between the two age categories, being greater in U12s in all cases compared with U13s. This may probably be due to the players having an inferior game level, resulting in more loss of the ball and therefore, more changes of possession, which would translate to a constant re-positioning of the players on the pitch, to respond to the game principles of opening up in attack whilst tightening defence [44]. Perhaps an inferior distribution of the younger players could also explain this, as they would have a more poorly adjusted collective behaviour [45], being drawn to the ball. However, when dimensions and player numbers per team were increased, so did this indicator in the U13s category. On the other hand, a higher W:R ratio in the U13s category (in the A300 dimensions) reflects a faster game pace in players of this age and, conversely, greater fatigue in the U12s [25]. With regard to the players’ maximum speed, the values were similar to those of other academies of elite junior players [37], where a peak speed of 24.2 ±0.2 Km h^-1^ was recorded for the U12s and 25.0 ±0.2 Km h^-1^ for the U13s. Although no significant differences were registered in V_max_ applied to LSGs, a tendency among the younger players (U12s) to reach higher peak speeds in A100 format was detected (irrespective of the number of players per team). However, it was the U13s who obtained a higher maximum peak speed in areas over 200 m^2^. It is probable that the space required for older players to reach maximum speeds could explain this fact [37].

Significant differences of %HR_mean_ were only observed in P11 in a pitch size of 200 m^2^ (A200), with the U12s achieving significantly lower values than the U13s. Despite there being no significant differences in the different ranges of heart rate intensity in the A100 format, as the number of players per team was increased along with the pitch size, the range of lower intensity heart rate (<75%HR_max_) was higher in U12s. To be more specific, it was in the 84–90%HR_max_ interval where significant differences in P11 for A300 were found, in favour of the U13 category. This could be due to an inferior collective competence in the U12s to respond to the demands of the game [45] in formats with many players and large areas. It also appears that the U13s are able to cope with more intense work rates throughout the period of the activity. Therefore, we could conclude that the time the U12s spent in each heart rate intensity zone is inferior in ranges of high heart rate intensity. Conversely, they remain longer in low heart rate intensity zones in formats where the absolute space is large (high number of players and high relative areas), which may suggest a physical limitation to the adequate development of their game dynamic.

With regard to locomotor activity, it is worth pointing out that, in absolute terms, the U13s registered a higher rate of movement in many of the analysed LSGs. A tendency emerged whereby, as size of space and number of players per team were increased, thereby producing larger absolute pitch size, these differences became more evident, reaching significance in U13s in the range of 13.0–16.0 Km h^-1^ in dimensions A200 and A300 for P11 and A300 for P9. Conversely, it was the U12s who covered a greater distance in the lower speed categories (<8.0 km h^-1^). This could be down to the physical profile of the players, being physically inferior at U12 and getting faster as they get older (U13>U12). Therefore, the results should be considered in relative terms in order to assess the physical ‘demand’ of each of the formats in relation to the potential of each player [25].

In relative terms, there was a similar profile of physical demands in both categories in the different formats studied. Firstly, it should be said that the players’ maximum speed values are similar to those of young elite players in other academies [37], where a peak speed for U12s of 24.2 ±0.2 Km h-1 was registered and of 25.0 ±0.2 Km h-1for U13s. As with the findings from studies carried out with senior players [15], the time spent in high speed ranges increased as pitch size (A100<A200<A300) and number of players per team (P17<P9<P11) were increased, which is explained in this study by a longer time spent in the range >60% of V_max_. There were hardly any differences between the categories for LSGs of 7 and 9 players, with a pitch size of 100 and 200 m^2^. However, in eleven-a-side matches (in any dimension) and 300 m^2^ pitch size (irrespective of player numbers), significant differences arose, reflecting a higher physical demand required from the younger players.

The main conclusions from this study can by summarised in various points. Firstly, modification of variables such as pitch size and number of players per team affects both the physical and physiological demands on young players, and what is more, it affects U12s and U13s differently. Secondly, in terms of methodology, it would appear to be recommendable to use relative values, i.e. those related to individual maximums, to assess the load borne when comparing the physical demand in players of different ages [26]. Thirdly, the results of this study would appear to suggest that there is a greater physical demand in U12 players compared with U13s in LSGs involving a larger relative space and higher number of players per team. Finally, when evaluating demand or the effects on players of modifying certain variables, attention should be paid to aspects which provide information about both the internal and external load resulting from the activity, thus allowing a more holistic assessment of the demands stemming from these. One possible application of the study could be that it is preferable not to propose football formats with dimensions of 300 m^2^ as sports modalities for the U12 category, irrespective of the number of players per team, not even with eleven-a-side, irrespective of relative dimensions per player.
